# Effects of COVID-19 Infection in Healthy Subjects on Cardiac Function and Biomarkers of Oxygen Transport, Blood Coagulation and Inflammation

**DOI:** 10.3390/v15081623

**Published:** 2023-07-25

**Authors:** Nadezhda G. Gumanova, Alexander U. Gorshkov, Natalya L. Bogdanova, Andrei I. Korolev

**Affiliations:** National Research Center for Preventive Medicine (NRCPM), Petroverigsky, 10, Building 3, 101990 Moscow, Russia; gumanova@mail.ru (N.G.G.); agorshkov@gnicpm.ru (A.U.G.); akorolev@gnicpm.ru (A.I.K.)

**Keywords:** COVID-19, echocardiography, left ventricular mass index, the ratio between the E and A waves, deceleration time of early mitral inflow, biomarkers

## Abstract

Background: The manifestations, severity, and mortality of COVID-19 are considered to be associated with the changes in various hematological parameters and in immunity. Associations of immunoglobulin G antibodies against severe acute respiratory syndrome-linked coronavirus (IgG-SARS)-positive status with cardiac function and hematological and biochemical parameters in apparently health subjects are poorly understood. Methods: The present cross-sectional study included 307 healthy volunteers (24–69 years of age; 44.8 ± 8.6 years; 80.4% men) and was initiated in 2019 before the COVID-19 pandemic. COVID-19 episodes were confirmed by detection of IgG-SARS against SARS-CoV-2 S1 RBD to reveal 70 IgG-SARS-positive and 237 negative participants. Numerous ultrasound characteristics were assessed by echocardiography, and 15 hematological and biochemical parameters were assayed in the blood. Descriptive and comparative analysis was based on the IgG-SARS status of the participants. Results: The left ventricular mass index, mitral ratio of peak early to late diastolic filling velocity or flow velocity across the mitral valve, and deceleration time of early mitral inflow were decreased (*p* < 0.05) in IgG-SARS-positive participants versus those in IgG-SARS-negative participants according to multivariate logistic regression analysis. Erythrocyte sedimentation rate and platelet count were slightly increased, and blood hemoglobin was decreased in IgG-SARS-positive participants compared with those in IgG-SARS-negative participants. Conclusions: LV filling, inflammation, blood coagulation, and hemoglobin appear to be influenced by COVID-19 infection in healthy participants. Our observations contribute to the definition of vulnerabilities in the apparently healthy subjects with long COVID-19. These vulnerabilities may be more severe in patients with certain chronic diseases.

## 1. Introduction

In 2023, the WHO Director-General determined that COVID-19 is an established and ongoing health issue that no longer constitutes a public health emergency of international concern. The consequences of COVID-19 infection are a potential problem for cardiovascular health all over the world [1]. Hospitalized patients with COVID-19 present with acute cardiac compromise, including acute heart failure (3–33%), cardiogenic shock (9–17%), myocardial ischemia or infarction (0.9–11%), left ventricular (LV) dysfunction (10–41%), right ventricular (33–47%) and biventricular dysfunction (3–15%), stress cardiomyopathy (2–5.6%), arrhythmias (9–17%), venous thromboembolism (23–27%), and arterial thrombosis secondary to virus-mediated coagulopathy (for review see [2]). Recent evidence suggest that diastolic dysfunction is also a common finding in COVID-19 hospitalized patients, with an incidence between 46% and 81% [3,4]. Information on healthy subjects with COVID-19 infection in anamnesis is very scarce. We have previously demonstrated that adenovirus infection may be an etiological factor of atrial fibrillation [5]. Thus, a viral infection may have harmful effects on cardiac function due to the mechanisms related to transcriptional and posttranslational modifications that disrupt the gap junctions, influence immune response, or mediate the development of cardiomyopathy during an active infection [6]. The information on cardiac dysfunction after COVID-19 in healthy subjects is limited. Echocardiography is a widely available method of assessment of the structural and functional characteristics of the heart. We aimed to compare the echocardiographic characteristics and the levels of routine blood markers in healthy participants recruited in a 2019–2021 study originally unrelated to COVID-19 [7]. The recruitment of this cohort was initiated before the pandemic and continued during the pandemic, and the data were used for comparative analysis of the groups of immunoglobulin G antibodies against severe acute respiratory syndrome-linked coronavirus (IgG-SARS)-positive and IgG-SARS-negative participants in the present study.

## 2. Materials and Method

### 2.1. Participants

Healthy volunteers (N = 307; 24 years of age and older) without prior cardiovascular disease or significant comorbidities were included in a cross-sectional study of microcirculation. Inclusion criteria were subjects over 18 years of age who volunteered to participate in the microcirculation study. The participants were recruited in 2019 (N = 80), 2020 (N = 129), and 2021 (N = 98) in National Research Center for Preventive Medicine (NRCPM), Moscow, Russia [7]. Evaluation of the participants included assessment of baseline cardiovascular characteristics by echocardiography, SCORE index calculation and biochemical parameters in the blood at the time of the recruitment visit.

The following exclusion criteria were applied: any acute inflammation, including oral or dental inflammation, at the enrollment date was considered an exclusion criterion; hematological diseases; left ventricular ejection fraction below 40%; diabetes mellitus; chronic kidney, liver, or heart failure; oncological diseases; mental illness; autoimmune diseases; any blood sugar lowering therapy; any cholesterol-lowering therapy; pregnancy; and lactation.

The protocol of the study was approved by the local Independent Ethics Committee of NRCPM; Protocol number: 01/01-2019 (12 February 2019) and was compliant with the guidelines of the Helsinki Declaration and WHO. All participants gave their written informed consent to participate in the study and to grant access to their personal data.

### 2.2. General Examination

Body weight was measured at a 0.1-kg precision using an electronic scale (Seca Ltd., Hamburg, Germany). Height was measured using a stadiometer to the nearest 0.1 cm (Seca Ltd.). The body mass index (BMI) was calculated according to the equation: weight/height^2^ (kg/m^2^).

### 2.3. Echocardiography

The data were collected at the visit date, including the size and shape of the heart, ventricular dimensions, pumping capacity, and location of tissue damage according to the standards for echo measurements of Intersocietal Commission for Accreditation of Echocardiography Laboratories (ICAEL) by a GE Vivid E95 ultrasound system with a 5.1 MHz transducer. Image postprocessing was done using EchoPAC software BT13 Viewing (GE Healthcare, Chicago, IL, USA) [8]. Two-dimensional echocardiography modes M or 2D and B were used, and pulse-wave and continuous-wave Doppler in the supine position were performed by a Vivid7 Dimension/Vivid 7 PRO echocardiography system (version 6.0.x, GE Healthcare, Munich, Germany). Cavity diameters and LV mass index were determined using the linear methods at the blood-tissue interface. LV systolic function was evaluated based on LV ejection fraction (LVEF) evaluated using the Simpson method and on LV global longitudinal strain (LVGLS). Abnormal LVEF was defined as <50%. Diastolic function, left atrium (LA) indexed volume, and other parameters were evaluated as recommended by current guidelines [9]. Abnormal diastolic function was defined as a mitral E/A ratio ≤ 0.8 and ≥2 in men and women [10]. An ambulatory 24-h blood pressure monitoring was used to evaluate systolic (SBP) and diastolic blood pressure (DBP) values by a Spacelabs Medical portable system (Heleige, Nurnberg, Germany). Office systolic and diastolic blood pressure was measured on the right arm after 5–10 min of quiet rest twice, and the average values were used for calculations.

### 2.4. Blood Sampling

Blood was withdrawn from the cubital vein after 12–14-h fasting on the date of the visit. The serum was obtained by centrifugation at 1000 g for 15 min at 4 °C. The serum and plasma samples were aliquoted and stored at −25 °C.

### 2.5. Blood Tests

Routine tests were performed in NRCPM using specific guidelines approved by Center for External Quality Control of Clinical Laboratory Testing of Russian Federation (www.fsvok.ru, (accessed on 10 September 2021)). Clinical blood tests were performed on an MEK-8222 K automatic hematology analyzer (Nihon Kohden, Saitama, Japan). Serum levels of alanine aminotransferase (U/L), aspartate aminotransferase (U/L), uric acid (mg/dL), creatinine (µmol/L), total cholesterol (mmol/L), high density lipoprotein (HDL)-cholesterol (mmol/L) after precipitation of apoB-containing low density lipoproteins (LDL), and triglycerides (mmol/L) were assayed using an Architect c8000 autoanalyzer and reagents from Abbot Diagnostics, Lake Forest, IL, USA. The level of LDL-cholesterol (mmol/L) was calculated according to the Friedwald equation in the samples with serum triglycerides below 4.5 mmol/L. C-reactive protein (CRP; mg/L) was assayed by high sensitivity quantitative immunoturbidimetric method enhanced with latex particles (universal range 0.3–350 mg/L; highly sensitive range 0.05–20 mg/L) using an Architect c8000 analyzer and reagents from Abbot Diagnostics. Plasma glucose level (mmol/L) was assayed by the hexokinase method using the same autoanalyzer and reagents from Abbot Diagnostics.

### 2.6. Detection of IgG Antibodies against SARS-CoV-2 S1 RBD

Enzyme immunoassay for qualitative detection of IgG antibodies against SARS-CoV-2 S1 RBD (IgG-SARS) was performed using an anti-SARS-CoV-2 ELISA E111-IVD kit (Mediagnost, Kusterdingen, Germany) by two-step enzyme-linked immunosorbent assay with recombinant SARS-CoV-2-S1 receptor binding domain (RBD) according to the manufacturer’s instructions as described elsewhere [7]. Deionized water (Aquasmart, Moskva, Russia) was used to dissolve the reagents. The samples were considered antibody-positive at >5-fold cut-off value or if the optical density was higher than 0.830. The samples with <3-fold cut-off value were considered antibody-negative. Samples from 3-fold to 5-fold cut-off or with the intermediate optical density values were considered borderline positive. The cut-off values were selected to ensure the exclusion of false positive results with a high probability. The assay was characterized by 95.55% sensitivity, 98.36% specificity, and 10.6% inter-assay variance [11].

### 2.7. Statistical Analysis

The data were analyzed using IBM SPSS Statistics 23 and Statistica software version 8.0 (StatSoft, Inc., USA). Some clusters of the data did not pass the Kolmogorov-Smirnov test for normality of distribution; therefore, we used non-parametric tests for all calculations. Continuous variables are presented as the mean ± SD, and categorical variables are presented in percentages. Sample size and power were estimated using the online calculator Sampsize https://sampsize.sourceforge.net/iface/s2.html#nm (accessed on 10 September 2021). Comparisons between the two groups were performed by Mann-Whitney U test. Chi-squared test, 2-sided Fisher’s exact test, and odds ratio (OR) with 95% confidential interval (95% CI) were used to evaluate the differences between the groups. In the case of categorical data, significance of OR was confirmed using Chi-squared Wald test. *p* values < 0.05 were considered significant.

## 3. Results

The present cross-sectional study comprised 307 volunteers from 24 to 69 years of age (44.8 ± 8.6; 80.4% men) enrolled in the microcirculation study in 2019–2021 [7]. All participants had no complaints for pre-existing cardiovascular diseases, thrombotic events or serious comorbidities. General, clinical, echocardiography, and biochemical characteristics are listed in Table 1. In general, the clinical and biochemical parameters varied within the normal range, indicating that the participants were apparently healthy. IgG-SARS were assayed in the blood samples of all participants. Only 20% of IgG-SARS-positive participants have reported a respiratory disorder before the enrollment and during 27–476 days of follow-up by phone survey by the endpoint of the study in 2022 [7].

The cohort was split into two groups according to the IgG-SARS status (positive or negative), and all parameters were compared between the groups using Mann-Whitney U test (Table 2). Three hematological and biochemical parameters were increased in IgG-SARS-positive participants (*p* < 0.05): ESR that may reflect the inflammation status, platelet count, and glucose levels. Six parameters were somewhat decreased in IgG-SARS-positive participants (*p* < 0.05): erythrocyte count, hemoglobin content, uric acid, creatinine, alanine aminotransferase, and aspartate aminotransferase (Table 2). The levels of 24-h SBP and DBP and 16 echocardiographic parameters representing the volume, mass, and velocity characteristics of the heart were decreased in IgG-SARS-positive participants (Table 2). Aortic valve regurgitation values were different between the groups (*p* < 0.05); however, the mitral, tricuspid, and pulmonary aortic valve regurgitation degrees were similar (Table 2 and Table 3). Surprisingly, the prevalence of the null degree of aortic valve regurgitation was 3.6-fold higher in IgG-SARS-positive subjects compared with that in IgG-SARS-negative participants (Table 3).

All parameters significantly different between the groups according to Mann-Whitney U test (*p* < 0.05) were included in the regression logistic models to assess independent impact of the covariates on the associations with IgG-SARS-positive or -negative status of the participants (Table 4 and Table 5). Three echocardiographic and three biochemical parameters were associated with IgG-SARS-positive status independently of other covariates according to the data of multivariate logistic regression (Table 4 and Table 5). Thus, left ventricular mass index (LVMI), mitral ratio of peak early to late diastolic filling velocity or flow velocity across the mitral valve (E/A), and deceleration time (DT) of early mitral inflow (E) were decreased (*p* < 0.05) in IgG-SARS-positive participants compared with those in IgG-SARS-negative participants (Table 2). Mitral valve regurgitation was similar in the two groups. LVMI is used as a marker of LV hypertrophy with excellent accuracy [12]. E/A is used to assess diastolic filling and is known as a predictor of mortality in middle-aged and elderly adults [13]. DT of early mitral inflow is considered a marker of diastolic LV chamber stiffness and is routinely measured during evaluation of LV diastolic function by Doppler echocardiography [14]. Thus, COVID-19 infection predominantly appears to adversely affect the LV function due to a decrease in various parameters.

Three biochemical parameters used in the logistic model were associated with IgG-SARS-positive status independently of other covariates (Table 5). ESR and platelet count were slightly increased, and hemoglobin content was decreased in the group with IgG-SARS-positive status compared with that in the IgG-SARS-negative group (Table 2). These parameters are considered the markers of inflammation, blood coagulation, and oxygen transport, respectively.

Thus, the data suggest that COVID-19 infection adversely affected cardiac function due to a decrease in LVMI and flow velocity across the mitral valve, transport of oxygen in the blood due to lowered content of hemoglobin [15], blood coagulation due to an increase in the platelet count, and inflammation reflected by an increase in ESR.

## 4. Discussion

Little is known about the degree of cardiovascular involvement after COVID-19 infection in healthy subjects. The data of the present study indicated that LVMI, E/A, and DT were decreased in SARS-positive participants independently of the covariates according to the results of logistic regression analysis. This finding is in agreement with the results of other authors indicating that various ventricular dysfunctions are manifested in more than 70% of subjects during the first 3 months after a COVID-19 episode [16]. Decreased ventricular function is more frequent in subjects with a moderate to severe episode, and subjects with persistent symptoms manifest a decrease in right ventricular function [16]. The results of the present study are consistent with the data of echocardiography obtained in a previous study of other authors, which demonstrated that basal-mid-apical index (BMAI) was increased in 70% of patients with persistent dyspnea recovered from COVID-19 [17]. Additionally, BMAI can be used as a predictor of LV thrombus in patients with systolic dysfunction [18]. The most common abnormalities are associated with low global longitudinal strain of the left and right ventricles according to a study of 595 participants (45.5 ± 14.9 years of age) in 10 institutions in Argentina and Brazil. Cardiovascular abnormalities after COVID-19 infection are usually mild, especially following a mild infection [1].

The LV filling is also characterized by mitral E/A. The E/A ratio is the simplest and most commonly used index to assess diastolic filling or evaluate the LV filling pressure. Individuals with low E/A ratios are considered to have impaired early diastolic relaxation [19,20]. Mitral E/A > 1.5 and E/A < 0.6 at baseline according to the data of Doppler echocardiography were associated with a 3- and 2-fold increase in cardiac mortality, respectively, in a population-based study in middle-aged and elderly adults [13]. The E/A ratio ≥ 1.5 reflects the restrictive filling, is a powerful predictor of heart failure in ambulatory patients with stable coronary artery disease [14], and is used to diagnose diastolic heart failure [21].

LV diastolic function is routinely characterized by DT of early mitral inflow. DT was decreased in the group of SARS-positive subjects in the present study. DT is considered a marker of diastolic LV chamber stiffness, and shortened DT after myocardial infarction is associated with worse cardiovascular outcome during follow-up [14].

The effect of COVID-19 on myocardial characteristics has been investigated in numerous studies. However, COVID-19 effects on thrombosis and blood coagulation in healthy subjects are poorly understood. COVID-19 infection is characterized by dysregulated thrombosis and coagulation that can increase mortality in patients [22]. The data of the present study indicated that platelet count was slightly increased in SARS-positive participants (*p* < 0.05); however, these changes were within normal limits. Platelets are fast responders to the presence of a pathogen and alert adjacent immune cells, contributing to thrombosis and intravascular coagulation. The presence of SARS-CoV-2 genome was detected in platelets from patients with COVID-19, which demonstrated an increased prothrombotic potential due to increased aggregation in the presence of stimulatory factors [22].

ESR was increased in the SARS-positive group in the present study. This observation is consistent with ongoing systemic inflammation in patients that can persist for several months after COVID-19 [23]. COVID-19 severity and mortality are known to be associated with specific changes in hematological characteristics and immunity. ESR and other hematological parameters were shown to be significantly increased in a cohort of 61 COVID-19-positive patients from Bangladesh [24]. A higher acute systemic inflammatory response was shown to be associated with a larger decrease in blood hemoglobin levels in a 55-year-old male patient admitted for a period of 28 days. The levels of CRP of 175 mg/L and higher are associated with a decrease in blood hemoglobin by 11 g/L compared with a decrease by only 6 g/L in subjects with CRP level below 4 mg/L [25]. Similar dynamics of hemoglobin were observed in a case-control study comprising 174 cases and 75 controls [23]. The data of the present study indicated that blood hemoglobin levels were decreased in SARS-positive participants by almost 11 g/L; however, the levels of CRP were similar in all participants. Thus, a decrease in hemoglobin levels after COVID-19 may persist longer than the signs of general inflammation.

## 5. Conclusions

The changes in the levels of LV filling, inflammation, blood coagulation, and blood hemoglobin were independently associated with IgG-SARS-positive status of healthy participants. The majority of subjects who are infected with COVID-19 recover within a few weeks. However, recurrent health problems can last for a long time after COVID-19 infection. These ongoing health problems are known as post-COVID-19 syndrome or long COVID-19. Long COVID-19 can affect some or all organs and may have a negative impact on daily life. Long COVID-19 is a new defined condition, and healthcare experts are still actively studying the patients. There is no specific treatment for this condition. Our observations contribute to the definition of vulnerabilities in the subjects with long COVID-19. These vulnerabilities may persist even in apparently healthy subjects and may require specific attention of the primary care physicians or specialists to ensure the lack of adverse development of the symptoms. Moreover, these vulnerabilities may be more severe in patients with certain chronic diseases.

## 6. Limitations

The present study did not reveal significant thrombotic events in the participants, which is one of the limitations. Thus, there are insufficient clinical data to support the conclusion on changes in blood coagulation in COVID-19 that is mainly based on increased platelets count. However, the studies of other authors reported considerable changes in coagulation-related events [22]. In the future, we plan to test additional markers of coagulation and inflammation processes to substantiate our conclusions.

## Figures and Tables

**Table 1:** General, echocardiographic, and biochemical characteristics of the total cohort (N = 307).

Parameters	Mean	S.D.
Age (years)	44.8	8.6
Height (cm)	175.2	8.4
BMI	27.2	4.3
SBP in office (mm Hg)	125.4	14.9
DBP in office (mm Hg)	82.3	9.9
SBP in 24-h monitoring (mm Hg)	123.4	12.9
DBP in 24-h monitoring (mm Hg)	80.5	9.1
SCORE (%)	1.85	2.3
Echocardiography (normal range)		
AoSV (2.9–4.5 cm)	3.3	0.4
LA (2.7–4.0 cm)	3.6	0.4
LA volume (37–94 mL)	51.4	13.9
LAVI (15–34 mL/m^2^)	25.6	5.7
LVIDd (3.5–5.6 cm)	4.9	0.5
LVIDs (2.0–4.0 cm)	2.7	0.4
LVEDV (90–150 mL)	105.1	26.5
LVESV (14–61 mL)	36.6	12.7
SV (50–100 mL)	69.0	16.2
CO (5–8 L)	4.7	1.1
LVEF (52–72%)	65.2	4.6
IVSd (0.6–1.0 cm)	1.0	0.2
LVPWd (0.6–1.0 cm)	0.9	0.1
LVM (67–224 g)	163.4	44.0
LVMI (43–115 g/m^2^)	80.9	16.9
Aortic valve opening amplitude (>1.5 cm)	2.1	0.2
RA volume (24–81 mL)	44.2	13.1
RVOT (<36 mm)	2.8	0.3
PA (15–25 mm)	2.0	0.3
sPAP (<36 mmHg)	22.3	4.6
IVC (0.97–2.26 cm)	1.8	0.2
E/A (0.8–2.0)	1.2	0.3
DT (<220 msec)	181.9	26.8
E/e′ (>6)	6.6	1.9
Epicardial fat thickness (0.9–13.5 mm)	4.8	4.2
Aortic valve regurgitation; degree 0, 1, 2 (%)	75.3; 23.4; 0.1	NA
Mitral valve regurgitation; degree 0, 1, 2 (%)	4.2; 94.5; 0.65	NA
Tricuspid valve regurgitation; degree 0, 1, 2 (%)	3.6; 95.4; 0.3	NA
Pulmonary aortic valve regurgitation; degree 0, 1, 2 (%)	2.9; 97.1; 0	NA
Routine biochemical markers (normal range)		
ESR (0–29 mm/h)	7.28	4.95
Erythrocytes (3.8–5.9 × 10^12^/L)	4.95	0.81
Hemoglobin (116–166 g/L)	149.88	18.48
Leukocytes (4–11 × 10^9^/L)	6.49	1.77
Platelets (150–450 × 10^9^/L)	223.24	47.95
Total cholesterol (<5.2 mmol/L)	5.54	1.05
HDL-cholesterol (>1 mmol/L)	1.41	0.42
LDL-cholesterol (<3.4 mmol/L)	3.49	0.93
Triglycerides (<1.7 mmol/L)	1.47	1.05
Glucose (3.9–5.6 mmol/L)	5.85	1.79
C-reactive protein (<3 mg/L)	2.23	5.32
Uric acid (2.7–8.5 mg/dL)	5.90	1.44
Creatinine (44–106 µmol/L)	80.57	14.68
Alanine aminotransferase (4–36 U/L)	26.18	17.81
Aspartate aminotransferase (8–33 U/L)	22.7	15.02

AoSV, aortic sinuses of Valsalva diameter; LA, left atrium anterior-posterior dimension; LAVI, left atrial volume index; LVIDd, left ventricular internal diameter at end-diastole; LVIDs, left ventricular internal diameter at end-systole; LVEDV, left ventricular end-diastole volume (biplane); LVESV, left ventricular end-systole volume (biplane); SV, stroke volume; CO, cardiac output; LVEF, left ventricular ejection fraction (biplane); IVSd, interventricular septum thickness at end-diastole; LVPWd, left ventricular posterior wall thickness at end-diastole; LVM, left ventricular mass; LVMI, left ventricular mass index; RA volume, right atrium volume; RVOT, right ventricular outflow tract proximal diameter; PA, main pulmonary artery diameter; sPAP, systolic pulmonary artery pressure; IVC, inferior vena cava diameter during expiration; E/A, early to late diastolic transmitral flow velocity; DT, deceleration time of early mitral inflow; E/e’, ratio between early mitral inflow velocity and mitral annular early diastolic velocity; ESR, erythrocyte sedimentation rate; HDL, high density lipoprotein; LDL, low density lipoprotein; NA, not applicable.

**Table 2:** Comparison of general, echocardiographic, and biochemical characteristics in the groups with IgG-SARS-negative and -positive status(*p* < 0.05 marked with color).

	IgG-SARS-Negative (N = 237)		IgG-SARS-Positive (N = 70)		Difference	*p*
Parameters	Mean1	S.D.	Mean2	S.D.	Mean1 − Mean2	
Age (years)	44.8	8.6	45.4	9.0	−0.6	0.67
Height (cm)	176.2	7.7	175.2	10.0	1.0	0.71
BMI (kg/m^2^)	27.4	4.2	26.8	4.7	0.7	0.12
SBP in office (mm Hg)	125.9	14.4	124.4	16.7	1.4	0.43
DBP in office (mm Hg)	82.9	9.4	80.5	11.6	2.4	0.05
SBP in 24-h monitoring (mm Hg)	124.9	12.8	118.8	12.2	6.1 *	0.001
DBP in 24-h monitoring (mm Hg)	81.2	9.0	78.4	9.3	2.8 *	0.02
SCORE (%)	1.9	2.5	1.6	1.7	0.3	
Echocardiography (normal range)						
AoSV (2.9–4.5 cm)	3.3	0.4	3.3	0.4	0.1 *	0.03
LA (2.7–4.0 cm)	3.6	0.4	3.5	0.3	0.1 *	0.02
LA volume (37–94 mL)	52.1	13.9	48.8	13.4	3.3 *	0.03
LAVI (15–34 mL/m^2^)	25.8	5.8	25.2	5.2	0.6	0.7
LVIDd (3.5–5.6 cm)	4.9	0.5	4.7	0.5	0.2 *	0.0001
LVIDs (2.0–4.0 cm)	2.7	0.4	2.8	0.4	−0.1	0.36
LVEDV (90–150 mL)	107.2	25.6	100.6	28.1	6.7 *	0.005
LVESV (14–61 mL)	37.4	10.3	35.1	16.5	2.2 *	0.008
SV (50–100 mL)	70.9	16.8	65.0	14.2	6.0 *	0.01
CO (5–8 L)	4.8	1.2	4.5	0.9	0.3	0.25
LVEF (52–72%)	65.1	4.1	65.3	6.0	−0.2	0.52
IVSd (0.6–1.0 cm)	1.0	0.2	1.0	0.2	0.0	0.34
LVPWd (0.6–1.0 cm)	0.9	0.1	0.9	0.1	0.1	0.20
LVM (67–224 g)	168.3	43.1	146.2	43.0	22.1 *	0.0001
LVMI (43–115 g/m^2^)	82.9	17.0	73.9	14.8	9.1 *	0.0001
Aortic valve opening amplitude (>1.5 cm)	2.2	0.3	2.1	0.2	0.1 *	0.0002
RA volume (24–81 mL)	45.3	12.7	40.5	14.0	4.8 *	0.01
RVOT (<36 mm)	2.7	0.3	22.3	5.0	−19.5	0.05
PA (15–25 mm)	2.0	0.3	2.8	0.3	−0.8	0.05
sPAP (<36 mm Hg)	22.6	4.2	21.4	5.7	1.2 *	0.01
IVC	1.8	0.2	1.8	0.3	0.0	0.1
E/A (0.75–1.5)	1.2	0.3	1.1	0.3	0.1 *	0.03
DT (<220 msec)	184.1	27.1	174.3	24.5	9.7 *	0.003
E/e′ (>6)	6.8	1.9	6.1	1.7	0.7 *	0.03
Epicardial fat thickness (0.9–13.5 mm)	4.8	4.6	4.7	2.5	0.1	0.9
Aortic valve regurgitation; degree 0, 1, 2 (%)	82.7; 27.2; 1.2	NA	90; 10; 0	NA	Table 3	
Mitral valve regurgitation; degree 0, 1, 2 (%)	5.5; 93.6; 0.8	NA	0; 100; 0	NA	NS	
Tricuspid valve regurgitation; degree 0, 1, 2 (%)	4.7; 95.7; 0.4	NA	0; 100; 0	NA	NS	
Pulmonary aortic valve regurgitation; degree 0, 1, 2 (%)	3.8; 96.2; 0	NA	0; 100; 0	NA	NS	
Routine biochemical markers (normal range)						
ESR (0–29 mm/h)	6.71	4.32	9.13	6.29	−2.42 *	0.005
Erythrocytes (3.8–5.9 × 10^12^/L)	4.97	0.47	4.88	1.47	0.09 *	0.00001
Hemoglobin (116–166 g/L)	152.27	17.97	141.86	18.01	10.41 *	0.00001
Leukocytes (4–11 × 10^9^/L)	6.46	1.55	6.58	2.37	−0.12	0.74
Platelets count (150–450 × 10^9^/L)	218.20	44.11	240.13	56.14	−21.93 *	0.018
Total cholesterol (<5.2 mmol/L)	5.57	0.97	5.42	1.29	0.15	0.13
HDL-cholesterol (>1 mmol/L)	1.40	0.43	1.46	0.37	−0.07	0.055
LDL- cholesterol (<3.4 mmol/L)	3.53	0.87	3.37	1.11	0.16	0.204
Triglycerides (<1.7 mmol/L)	1.50	1.07	1.39	0.99	0.10	0.1
Glucose (3.9–5.6 mmol/L)	5.84	0.75	5.86	3.54	−0.02 *	0.00001
C-reactive protein (<3 mg/L)	2.00	3.17	3.02	9.47	−1.02	0.9
Uric acid (2.7–8.5 mg/dL)	6.01	1.30	5.53	1.80	0.48 *	0.002
Creatinine (44–106 µmol/L)	82.02	13.18	75.78	18.09	6.24 *	0.002
Alanine aminotransferase (4–36 U/L)	26.99	17.38	23.51	19.04	3.48 *	0.002
Aspartate aminotransferase (8–33 U/L)	23.55	16.34	19.86	8.92	2.69 *	0.004

For abbreviations, see legend to Table 1. *p* values according to Mann-Whitney U test, * *p* < 0.05.

**Table 3:** Aortic valve regurgitation in IgG-SARS-positive and -negative participants.

Aortic Valve Regurgitation	IgG-SARS-Negative (N = 237)	IgG-SARS-Positive (N = 70)	Odds Ratio; 95% CI	*p*
Degree 0, N	169	63	3.6; 1.5–8.3	0.0024
Degrees 1 and 2, N	68	7		

**Table 4:** Multivariate logistic regression analysis of associations of independent variables with IgG-SARS-positive status(*p* < 0.05 marked with color).

Parameters in the Model	B	S.E.	Wald	*p*	Exp(B)	95% CI for EXP(B)	
						Lower	Upper
SBP in 24-h monitoring	−0.06	0.03	3.79	0.05	0.94	0.89	1.00
DBP in 24-h monitoring	0.03	0.04	0.58	0.45	1.03	0.95	1.11
AoSV	0.55	0.69	0.64	0.43	1.73	0.45	6.67
LA	1.68	0.78	4.71	0.11	5.37	1.18	24.49
LA volume	0.01	0.02	0.24	0.63	1.01	0.97	1.05
LVIDd	−0.13	0.65	0.04	0.84	0.88	0.25	3.15
LVEDV	−0.02	0.04	0.16	0.69	0.99	0.91	1.06
LVESV	−0.01	0.05	0.01	0.92	1.00	0.91	1.09
SV	−0.01	0.04	0.02	0.88	0.99	0.92	1.08
LVM	0.01	0.01	0.54	0.46	1.01	0.98	1.04
LVMI	−0.07	0.03	4.47	0.03	0.93	0.88	1.00
Aortic valve opening amplitude	0.38	1.12	0.11	0.74	1.46	0.16	13.21
RA volume	−0.01	0.02	0.15	0.70	0.99	0.96	1.03
sPAP	−0.01	0.04	0.11	0.74	0.99	0.92	1.06
E/A	−1.60	0.75	4.54	0.03	0.20	−1.60	0.75
DT	−0.02	0.01	5.13	0.02	0.97	−0.02	0.01
E/e′	0.03	0.12	0.05	0.82	1.03	0.82	1.29

For abbreviations, see legend to Table 1.

**Table 5:** Nominal regression analysis for IgG-SARS-positive participants and various parameters based on likelihood ratio tests(*p* < 0.05 marked with color).

Parameters in the Model	Model-Fitting Criteria; −2 Log Likelihood of Reduced Model	Likelihood Ratio Tests	
		Chi-Squared	*p*
ESR	279.81	4.23	0.04
Erythrocytes	275.74	0.16	0.68
Hemoglobin	280.79	5.21	0.02
Platelets	280.69	5.11	0.02
Glucose	275.80	0.22	0.63
Uric acid	275.58	0.003	0.95
Creatinine	276.63	1.06	0.30
Alanine aminotransferase	277.55	1.97	0.16
Aspartate aminotransferase	278.56	2.98	0.08

The value of the Chi-squared statistic describes the differences in −2 log-likelihoods between the final model and a reduced model. The reduced model is formed by omitting an effect from the final model. The null hypothesis is that all parameters of an effect equal 0. Pseudo R-squareds: Cox and Snell 0.12; Nagelkerke 0.18; and McFadden 0.12.

## Data Availability

Data is unavailable due to privacy or ethical restrictions.
